# Tail regeneration reduction in lizards after repetitive amputation or cauterization reflects an increase of immune cells in blastemas

**DOI:** 10.24272/j.issn.2095-8137.2018.050

**Published:** 2018-07-06

**Authors:** Lorenzo Alibardi

**Affiliations:** 1 Comparative Histolab Padua, Department of Biology, University of Bologna, Bologna 40126, Italy

**Keywords:** Lizard, Tail, Repetitive regeneration, Blood, Immune cells, Ultrastructure

## Abstract

Lizards are key amniote models for studying organ regeneration. During tail regeneration in lizards, blastemas contain sparse granulocytes, macrophages, and lymphocytes among the prevalent mesenchymal cells. Using transmission electron microscopy to examine scarring blastemas after third and fourth sequential tail amputations, the number of granulocytes, macrophages, and lymphocytes increased at 3–4 weeks in comparison to the first regeneration. An increase in granulocytes and agranulocytes also occurred within a week after blastema cauterization during the process of scarring. Blood at the third and fourth regeneration also showed a significant increase in white blood cells compared with that under normal conditions and at the first regeneration. The extracellular matrix of the scarring blastema, especially after cauterization, was denser than that in the normal blastema and numerous white blood cells and fibroblasts were surrounded by electron-pale, fine fibrinoid material mixed with variable collagen fibrils. In addition to previous studies, the present observations support the hypothesis that an increase in inflammation and immune reactions determine scarring rather than regeneration. These new findings verify that an immune reaction against mesenchymal and epidermal cells of the regenerative blastema is one of the main causes for the failure of organ regeneration in amniotes.

## INTRODUCTION

The regenerating tail of lizards is an outstanding and unique case of massive organ regeneration in amniotes, i.e., fully terrestrial vertebrates (Alibardi, 2010a; Bellairs & Bryant, 1985; Fisher et al., 2012; Gilbert et al., 2013). The process of tail regeneration in lizards is a uncommon event given the lack of any organ regeneration in other amniotes. During regeneration, numerous tissues are reformed and combined into a functional tail, albeit simplified in comparison to the original. Therefore, lizards are critical vertebrates for analyzing successful versus unsuccessful tissue and organ regeneration in amniotes, with potential consequences for mammalian regeneration (Alibardi, 2010a, 2014; Gilbert et al., 2013; Lozito & Tuan, 2017). Rare cases of anomalous regeneration have been recorded in relation to parasitic infection resulting in impaired health conditions (Oppliger & Clobert, 1997) or following intense damage accompanied by severe inflammation (Baffoni, 1950). Tail regeneration can be altered or inhibited under experimental manipulations such as cauterization, wounding the regenerating blastema, or removing the apical epidermal peg, thus resulting in scarring (Alibardi, 2010a, 2013, 2014). Therefore, normal regeneration in lizards depends on processes that control inflammation but can be interrupted by the above treatments.

Inflammation is a typical cell and humoral response elicited by traumatic mechanical (cuts, amputation), chemical (irritants, toxins), or physical (heat, cold, radiation) stimuli to tissues. Inflammation limits tissue and organ plasticity in different models of vertebrates (Leavitt et al., 2016; Mescher et al., 2013; Montali, 1988). Invasion of the regenerating tail blastema from immune cells, granulocytes, macrophages, and lymphocytes is poorly known in lizards, and based only on qualitative observations (Alibardi, 1994, 2010b, 2013, 2016; Alibardi & Sala, 1988). These previous ultrastructural and immunohistochemical studies have indicated that the scarring limb undergoes strong invasion from hematogenous cells, in particular mononucleate cells (macrophages and lymphocytes).

The importance of clarifying the role of the immune system in regenerative plasticity in amniotes was confirmed recently based on a transcriptome study comparing the regenerating tail blastema to that present in scarring limbs of the same lizards (Vitulo et al., 2017). The study indicated that inflammation-eliciting genes were unchanged but immune-genes were strongly down-regulated in the tail blastema, whereas inflammatory genes were up-regulated but immune-genes remained unchanged in the limb of the same animals.

The regenerating blastema therefore appears to function as an immune-privileged/suppressed organ, like the developing tail or limb, and the presence of immune cells under normal tail regeneration is likely limited and does not influence the process. Previous electrophoretic and hematological studies have indicated that the number of lymphocytes/macrophages and the gamma-globulin fraction tend to increase when tail regeneration is reduced or inhibited (Alibardi, 2014). However, the specific production of anti-regenerative antibodies or lymphocytes has not been demonstrated, and therefore the increase in the number of immune cells and gamma globulins might simply reflect a general state of inflammation. To date, no studies have measured the number or type of white blood cells, especially macrophages and lymphocytes, invading the blastema and circulating in the blood during manipulations that stimulate scarring (Alibardi, 2013, 2014). The present ultrastructural study aimed to investigate the hypothesis that the significant increase in white blood cells, in particular macrophages and lymphocytes, likely exerts an immunological reaction against blastema cells induced to form scarring fibroblasts instead of a mesenchymal mass of cells.

## MATERIALS AND METHODS

### Repetitive tail amputations

Five adult wall lizards (*Podarcis muralis*) of both sexes were utilized in the present study; they all survived and were eventually released in the wild. All experiments followed Italian regulations on animal care and handling (art. 5, DL 116/92). The lizards were left for 20 min at 4–6 °C to induce hypothermic numbness, and the tail was amputated with a razor blade at about the second to third proximal. The surface of the stump was kept wet and warm with the application of a small drop of Ringer fluid, and the extravasating blood from the stump was collected with a Pasteur’s pipette (30–50 μL) into a small 0.5-mL Eppendorf vial until no further bleeding was observed. A fixative consisting of 0.2 mL of 4% paraformaldehyde solution in 0.1 mol/L phosphate buffer at pH 7.4 was rapidly added to the blood samples for 5 min.

The Eppendorf vials were centrifuged for 5 min at 10 000 r/min to form a pellet, and fixation lasted about 8 h. From the same lizards, a 2-mm portion of the normal stump of the detached tail was fixed in the same fixative to represent normal controls. When the blastema of the first regeneration was 2–3 mm in length and coniform in shape, it was cut at the base and the blastema with extravasating blood was collected as above from the five animals, representing blastema and blood samples of the first regeneration. The operation was repeated in the following weeks to collect blastema and blood samples of the second, third, and fourth regeneration. As controls, three lizards were left to regenerate their tails normally. All outgrowths and collected blood samples were fixed as above, dehydrated, and embedded in Lowcryl resin under UV exposure for 2 d at 4 °C.

### Tail cauterization

Tail cauterization was performed on three lizards with regenerating blastemas 2–3 mm in length. Using a hot plate, a laminar spatula was heated for 10 s, then applied three times for 1–2 s to the tip of the blastema, scolding it without apparent external damage to the skin (i.e., no bleeding or cutting). The animals were left for 5–6 d in a cage, with the blastema then collected and fixed after growing ceased. The cauterized blastemas were cut in half, fixed in 4% paraformaldehyde as above and dehydrated in ethanol; one half was then embedded in wax (for other studies) and the other half was embedded in plastic and transparent resin Lowcryl.

### Preparation for histology and electron microscopy

Lowcryl-embedded blastemas were sectioned along the longitudinal plane using an ultramicrotome and were collected on glass slides, stained with 1% toluidine blue, and observed under a light microscope for histological analysis. The embedded blood samples (pellets) were also sectioned with an ultramicrotome. From regions of interest, thin 50–90-nm sections were collected on copper grids for transmission electron microscopy (TEM) study. The sections were stained for 45 min in 1% uranyl acetated water solution and for 5 min in lead citrate according to routine methods, then observed under a Zeiss C10 electron microscope operating at 60 kv. Images were acquired using a digital camera incorporated in the electron microscope.

Two regenerative blastemas (3 mm in length) were prepared for the Gomori reaction to reveal acid phosphatases, typical markers used to identify lysosome granules in white blood cells (Troyer, 1980), under electron microscopy. Tissues were fixed at 0–4 °C in 0.25% glutaraldehyde and 4% paraformaldehyde in 0.12 mol/L phosphate buffer at pH 7.4. The tissues were then cut with a vibratome to collect 50–70-μm sections, which were incubated for 45 min in 0.5 mol/L acetate buffer at pH 4.8 and then in Gomori-lead medium for 90 min at 27–30 °C for the detection of acid phosphatases (Troyer, 1980). After washing in acetate buffer for 30 min, the sections were rinsed in distilled water for 5 min, post-fixed in 1% osmium tetroxide for 1 h, and then dehydrated and embedded in EPON resin. Thin sections obtained using an ultramicrotome were collected on copper grids and observed under a Zeiss C10 electron microscope as above.

### Cell counts during successive amputations and after cauterization

Based on the relative percentages of cells in different stages of regeneration, two approaches were used to compare the relative variations in white blood cells present in blastemas or in blood following repetitive amputations of the tail or its cauterization. Firstly, the numbers of white blood cells (granulocytes and agranulocytes) were counted from whole TEM thin sections of regenerative blastemas at the first and fourth regeneration, and in cauterized blastemas. The number of detected white blood cells in each case was indicated as a percentage out of 400–500 blastema (mesenchymal) cells counted in blastemas at the first and fourth regeneration and one week after cauterization. Secondly, the blood samples were examined and the number of white blood cells, indicated as a percentage out of 700–900 red blood cells, was counted in the blood pellet sections collected from lizards in normal, first, third, and fourth regenerations. The number of TEM sections (*n*) for each individual, as well as the mean, standard deviation (mean±*SD*), and statistical significance using one-way ANOVA comparing variations in different stages, are reported in the text. Statistical significance was set at *: *P*<0.05, **: *P*<0.01, or ***: *P*<0.001.

## RESULTS

### Gross and general microscopy observations

**Figure 1:**
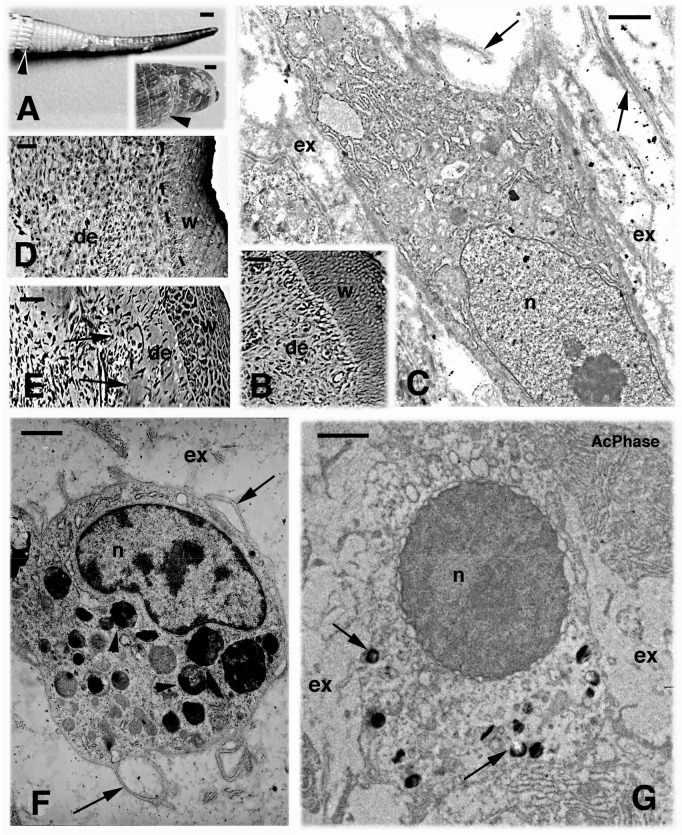
Gross aspects of regenerated tail versus scarring tail outgrowth (A) and microscopy images of areas of the regenerating tail (B–G)

The three lizards allowed to regenerate their tails normally produced long and scaled regenerated tails in about 30 d following amputation (Figure 1A). Conversely, compared with first and second regeneration blastemas, some third and most fourth regeneration blastemas appeared as short and hard scarring outgrowths. These outgrowths showed irregular scalation patterns and were 2–3 mm in length (inset in Figure 1A).

A: Largely scaled, regenerated tail at 30 d post-amputation. Arrowhead indicates transition point with stump of original tail. Scale bar: 1 mm. Inset shows a tail outgrowth at fourth regeneration (arrowhead on amputation region). Scale bar: 0.5 mm. B: Histology of a first regeneration blastema. Scale bar: 10 μm. C: Electron microscopy view of first regeneration blastema cells. Arrows indicate collagenous fibrils with amorphous matrix. Scale bar: 0.5 μm. D: Histology of a fourth regeneration blastema. Scale bar: 10 μm. E: Histology of a blastema at 6 d post-cauterization, showing large masses of pale extracellular material (arrows) underneath wound epidermis. Scale bar: 10 μm. F: Macrophage with numerous secondary lysosomes (arrowheads) and filopodia (arrows) within a first regeneration blastema. Scale bar: 1 μm. G: Macrophage within the apical region of a first regeneration blastema with acid phosphatase positive lysosomes (arrows). Scale bar: 1 μm. de: dermis; ex: extracellular matrix; n: nucleus; w: wound epidermis.

The normal regenerating tail blastema contained a homogenous mass of mesenchymal cells and fibroblasts localized underneath a multilayered wound (regenerating) epidermis (Figure 1B). Electron microscopy showed that these fibroblast-mesenchymal cells possessed a variable number of narrow endoplasmic cisternae and euchromatic nuclei with large nucleoli. The cells were surrounded by a loose extracellular space containing sparse amorphous material (hyaluronic acid) and scarce unbanded collagen fibrils (Figure 1C).

The inner tissues present in the regenerating outgrowths at the fourth regeneration displayed less regular connective tissue and irregular bundles of extracellular (collagenous) fibrils located among fusiform fibroblasts, especially in the dermis (Figure 1D). In cauterized blastemas, extracellular fibrils or masses of fibrin material were present among fusiform fibroblasts and blood vessels (Figure 1E).

### Electron microscopy and count variations at first and fourth regenerations

**Figure 2:**
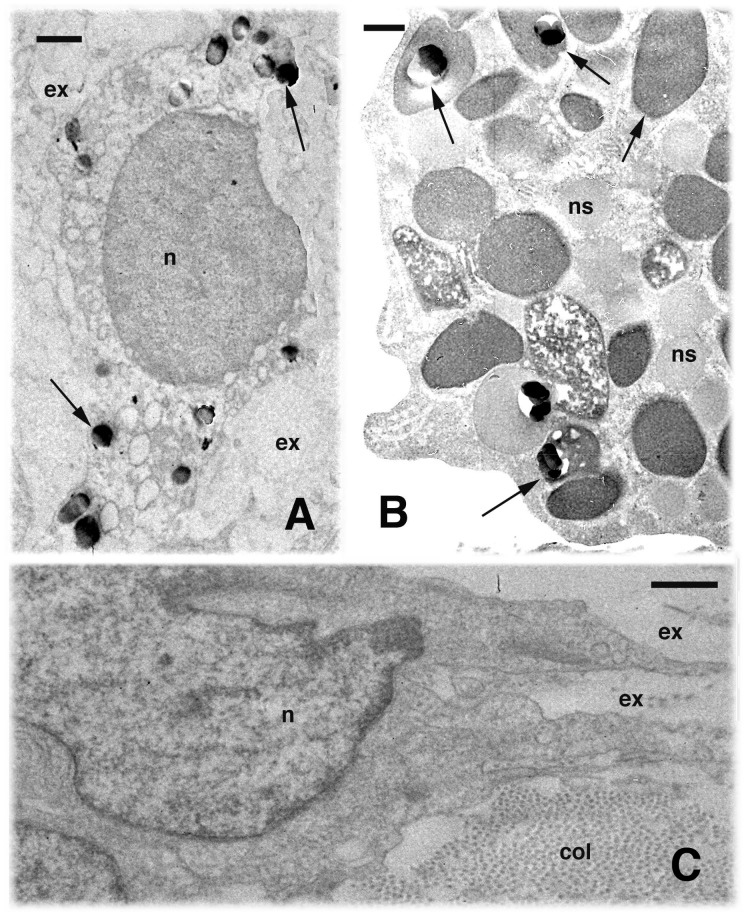
Electron microscopy view of white blood cells in a first regeneration blastema (A, B) and a fourth regeneration fibroblast (C)

The normal blastema (first regeneration) contained loose connective tissue with many mesenchymal cells and sparse macrophages (Figure 1C, F). The Gomori reaction showed that macrophages in apical or more proximal regions of the blastema contained acid phosphatase positive lysosomes (Figure 1G, Figure 2A). In the regenerating outgrowths following the fourth regeneration, electron microscopy revealed frequent macrophages and granulocytes containing lysosomes in various stages of digestion (Figure 1F). In the macrophages, lysosomes were acid phosphatase-positive (Figure 1G, Figure 2A), and in the granulocytes some granules appeared positive for the enzyme as well (Figure 2B). Among the fibroblasts present in the outgrowth, numerous collagen fibrils were seen, and the extracellular spaces were reduced to form dense connective tissues with respect to that of the normal blastema (Figure 2C).

A: Macrophages with acid phosphatase positive granules (arrows) in more proximal area of the blastema. Scale bar: 0.5 μm. B: Granulocyte with some acid phosphatase positive granules (arrows). Scale bar: 0.25 μm. C: Fibroblast in contact with large bundle of collagen fibrils. Scale bar: 1 μm. col: collagen fibrils; ex: extracellular matrix; n: nucleus; ns: non-specific granules.

**Figure 3:**
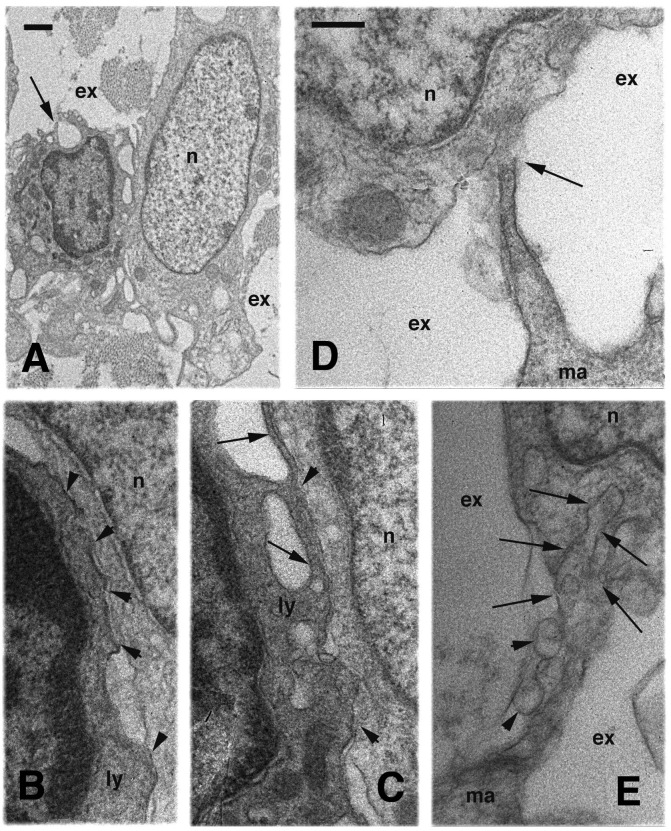
White blood cells present in a fourth regeneration blastema

**Figure 4:**
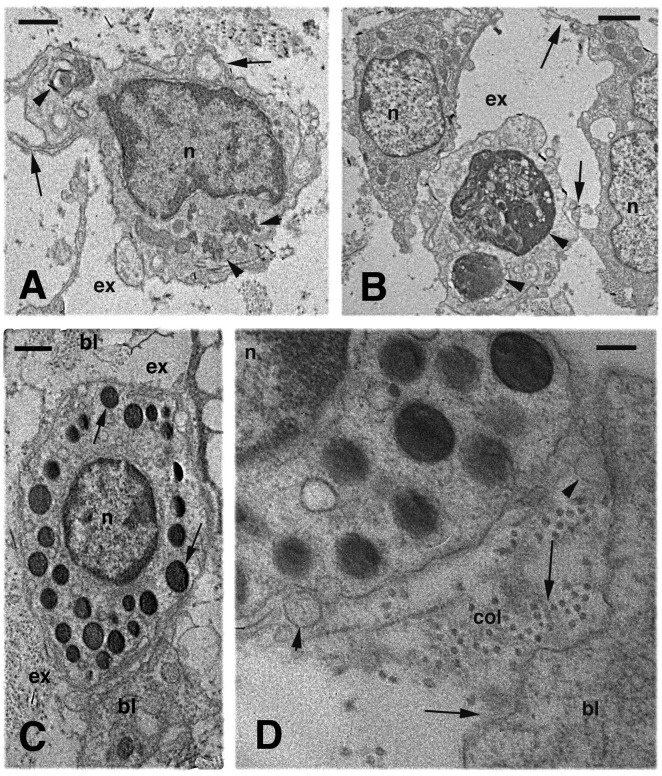
Electron microscopy view of white blood cells within a fourth regeneration blastema

**Figure 5:**
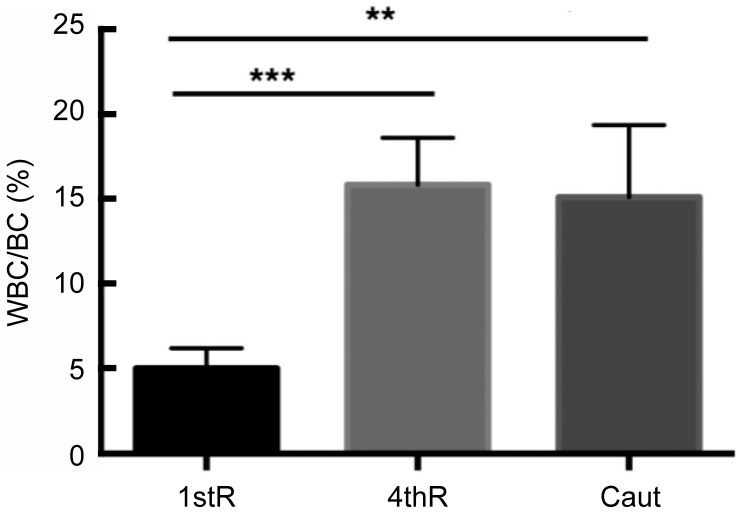
Statistical values and significance of variations in white blood cells/blastema cells in first regeneration (1stR) compared to fourth regeneration (4thR) or cauterized (Caut)

Furthermore, lymphocytes appeared relatively frequently among cells of the blastema in outgrowths at the fourth regeneration (Figure 3, Figure 4). Cells recognized as lymphocytes were smaller than blastema cells and showed a more electron-dense cytoplasm compared with the fibroblasts, with variable perinuclear chromatin. Large cytoplasmic regions of the lymphocytes contacted the membrane of the blastema cells (Figure 3A–C) or occurred through their thin elongation (Figure 3D, E). In some cases, the activated lymphocytes presented a very irregular surface with typical filopodia, as in the macrophages (Figure 4A). In these cases, their exact identification was doubtful, and therefore in our quantitative counts we considered these cells collectively as agranulocytes (see Figure 5). We considered true macrophages only for larger cells exceeding 10 μm. Other cells with a paler and vesicular cytoplasm, but containing large phagosomes or cell remnants, were considered as dying phagocytes (Figure 4B). The more frequent granulocytes encountered among the connective tissues of the outgrowths were represented by basophilic cells. These cells were recognized by their numerous and large globular granules and by their roundish, non-polymorphic nuclei, as observed for neutrophilic granulocytes (Figure 4C). These cells were in close contact with the blastema cells, although some amorphous material and collagen fibrils were often seen between the two cell types (Figure 4D). Small vesicles (0.1–0.2 μm in size) were also seen in these regions, though their origins remained unclear from our static images.

A: Mesenchymal fibroblast close to darker agranulocyte (arrow). Scale bar: 1 μm. B: Large appositional area (arrowheads) between lymphocyte (darker) and blastema cells (paler fibroblast). Scale bar: 0.25 μm. C: Contact area with filopodia (arrows) localized between a lymphocyte and fibroblast. Scale bar: 0.25 μm. D: Macrophage filopod contacting (arrow) mesenchymal cell. Scale bar: 0.25 μm. E: Filopod from a macrophage contacting or invading (arrows) a blastema cell. Arrowheads point to seemingly extracellular vesicles. Scale bar: 0.25 μm. ex: extracellular matrix; ly: lymphocyte; ma: macrophage; n: nucleus.

A: Agranular electron-dense cell with filopodia (arrows) and cytoplasmic dense bodies (arrowheads, likely lysosomes). Scale bar: 1 μm. B: Pale cell with two large phagosomes (arrowheads) near a cell with filopodia (arrows). Scale bar: 1 μm. C: Basophil granulocyte (arrows show large dense granules) detected among blastema cells. Scale bar: 1 μm. D: Narrow extracellular space present between a basophil and blastema cell, where collagen fibrils and amorphous material are present (arrows). Arrowheads point to apparent extracellular vesicles. Scale bar: 0.25 μm. bl: blastema (mesenchymal) cell; col: collagen fibrils; ex: extracellular space; n: nucleus.

**: *P*<0.01; ***: *P*<0.001.

The number of white blood cells present in first regeneration blastemas, out of 400–500 blastema cells, compared to the number of white blood cells in fourth regeneration blastemas, is presented in Figure 5. Results showed an almost three-fold increase in white blood cells at the fourth regeneration (mean 15.9), especially agranulocytes, compared to that at the first regeneration (mean 5.0). This value was significant according to the ANOVA test (Table 1, Figure 5).

**Table 1:** Percentages of white blood cells in first regenerative blastema compared with those present in fourth regenerative blastema and 6 d after blastema cauterization

	1st reg	4th reg	Cauterized
*n*	5	4	3
G (%)	45.2	28.8	40.2
A (%)	54.8	71.2	59.8
WBC (%)	5.0±1.5	15.9±1.6	15.1±1.8

*n*: Number of samples. G: Granulocytes (heterophils-neutrophils, basophils, and eosinophils). A: Agranulocytes (monocytes and lymphocytes). WBC: White blood cells.

### Electron microscopy and count variations in cauterized outgrowths

**Figure 6:**
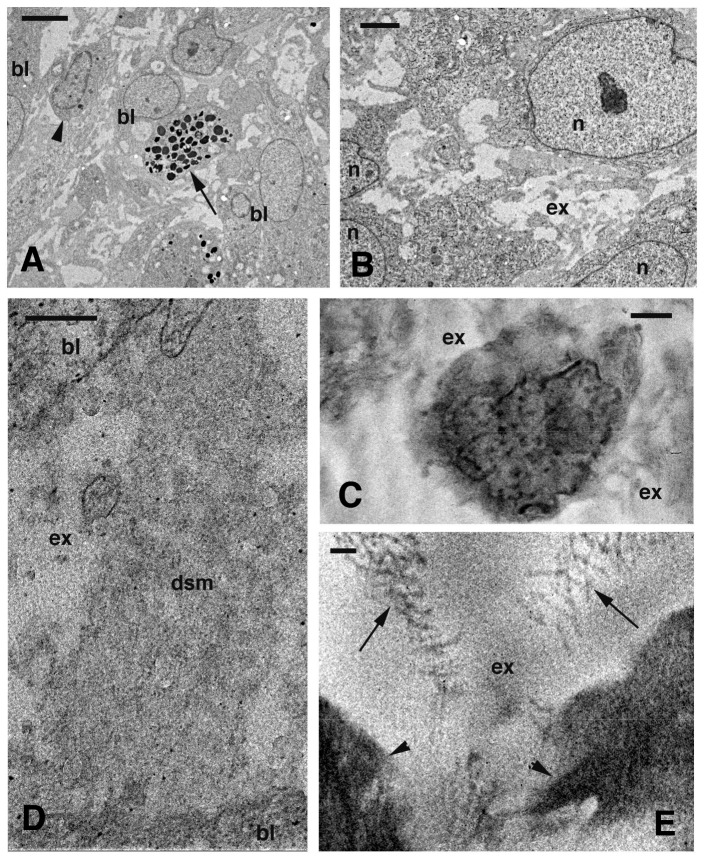
Electron microscopy view of blastema 6 d after cauterizatio

After cauterization, the blastema stopped its growth. Among blastema cells (fibroblasts), a reduced extracellular matrix was present in comparison to the normal blastema (Figure 6A, B). White blood cells were more frequently encountered than in the normal blastema at the first regeneration and appeared as agranulocytes (macrophages-lymphocytes) and granulocytes with large granules, likely basophils. Quantification indicated a three-fold increase (mean 15.1, see Figure 5) in white hematic cells in the blastema at 6 d post-cauterization compared with the number of white cells present in the blastemas in the first regeneration (mean 5.0, see Figure 5). This value was significant according to the ANOVA test (Table 1, Figure 5).

A: Basophil granulocytes (arrow) and macrophages (arrowhead) present among blastema cells. Scale bar: 2 μm. B: Electron-pale extracellular matrix present among blastema cells. Scale bar: 1 μm. C: Dark lymphocyte within the electron-pale extracellular exudate. Scale bar: 1 μm. D: Dense amorphous material present within the extracellular pale exudate. Scale bar: 0.25 μm. E: Denser fibrillar material (arrows) embedded in paler exudate between two dark cells (arrowheads). Scale bar: 0.2 μm. bl: blastema cells; dsm: dense extracellular and largely amorphous material; ex: extracellular space; n: nucleus.

A pale and finely granular material, resembling a plasma-fibrinous exudate, was present in the extracellular matrix (Figure 6C–E). Denser coarse filaments or fibrils of 30–40 nm and of unknown nature but resembling fibrin filaments (Odland & Ross, 1968), were also embedded in the exudate. These fibrils were irregularly distributed within the extracellular matrix.

### Electron microscopy of blood cells in normal, first, third, and fourth regeneration

**Figure 7:**
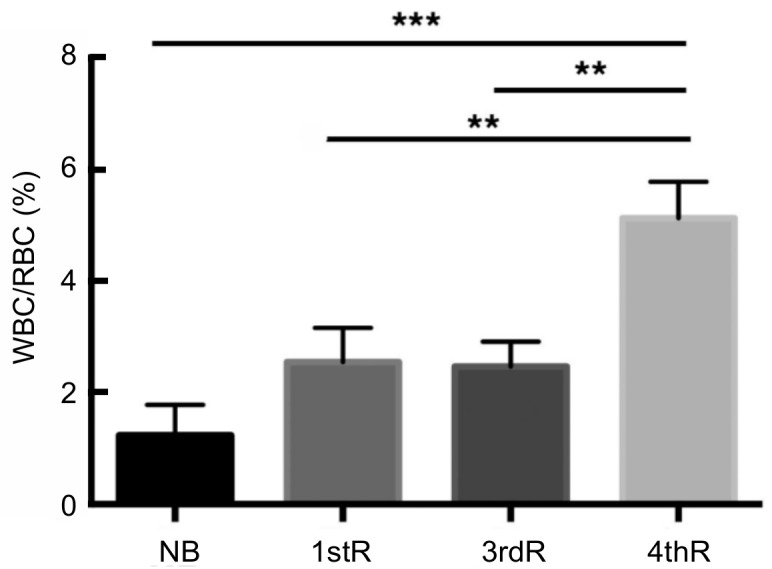
Statistical values (ANOVA test) and significance of variations in white blood cells/erythrocytes in normal blood (NB), first regeneration (1stR), third regeneration (3rdR), and fourth regeneration (4thR)

The blood cells derived from pellets of normal blood samples and after the first, third, and fourth regeneration demonstrated a significant 3–4-fold increase in white blood cells in general (1.2% in normal blood versus 5.1% in blood at the fourth regeneration) (Table 2, Figure 7). The value was not significant between normal and first regeneration or normal and second regeneration but was significant between first and second regeneration versus fourth regeneration and highly significant between normal and fourth regeneration (Figure 7).

**Table 2:** Percentages of white blood cells in the blood of normal lizards and in first, third, and fourth regeneration

	Normal	1st reg	3th reg	4th reg
*n*	3	4	3	3
G (%)	56.7	56.2	58.0	54.9
A (%)	43.3	43.8	42.0	45.1
WBC (%)	1.2±0.45	2.1±0.41	2.5±0.37	5.1±0.53

*n*: Number of samples. G: Granulocytes (heterophils-neutrophils, basophils, and eosinophils). A: Agranulocytes (monocytes and lymphocytes). WBC: White blood cells.

**: *P*<0.01; ***: *P*<0.001.

**Figure 8:**
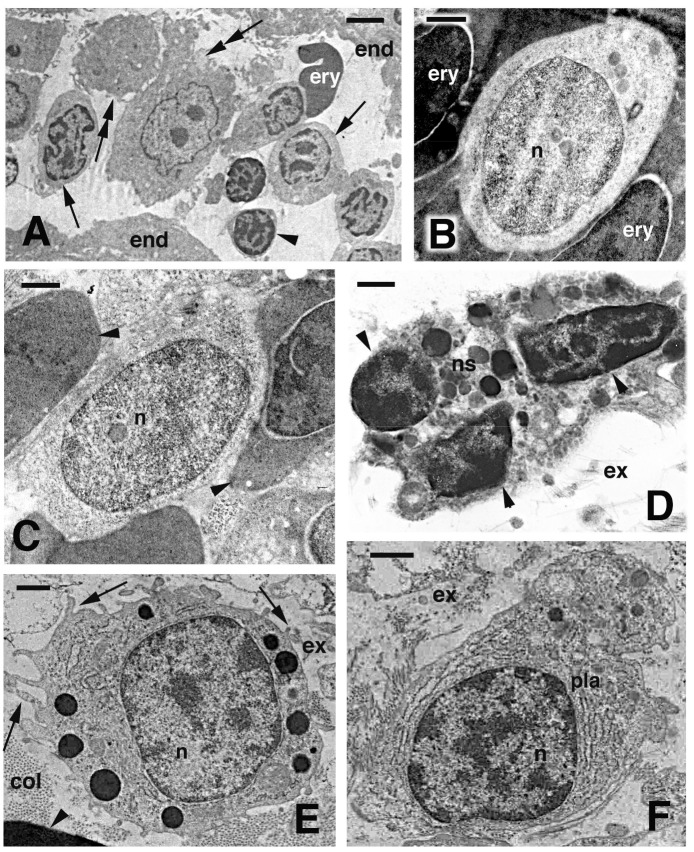
>Electron microscopy view of blood cells present in blood vessels within a blastema (A, D–F) or isolated in blood pellets (B, C) at the fourth regeneration

White blood cells in both pellets and blood vessels in the blastema were mainly represented by granulocytes (especially basophils), though macrophages and lymphocytes were also seen (Figure 8A). Numerous cells were likely lymphoblasts, characterized by a round shape and pale cytoplasm, and containing sparse free ribosomes and a nucleus featuring a more diffuse (unpacked) chromatin than that of small lymphocytes, macrophages, or granulocytes (Figure 8B–E). Completely differentiated plasma-cells were only found occasionally in blood pellets sections and within vessels in the blastema or in scarring outgrowths (Figure 8F).

A: In this large capillary, most lymphocytes (arrowheads), lymphoblasts (arrows), and monocytes (double arrows) are seen. Scale bar: 2 μm. B: Pale lymphoblast detected among erythrocytes. Scale bar: 1 μm. C: Lymphoblast present among erythrocytes. Scale bar: 1 μm. D: Polymorphonucleate granulocyte (arrowheads indicate three lobes of the nucleus). Scale bar: 1 μm. E: Mastocyte (basophil?) with filopodia (arrows). Scale bar: 1 μm. F: Plasma cell within blastema. Scale bar: 2 μm. col: collagen bundle; end: endothelial cell; ex: extracellular matrix; ery: erythrocyte; n: nucleus; ns: non-specific granules; pla: plasma-cell.

## DISCUSSION

This ultrastructural study demonstrated a significant increase in white blood cells, about three times higher than that observed during the first regeneration, in the cauterized and fourth repetitive regeneration blastemas, confirming previous studies (Alibardi, 2010a, 2010b, 2013, 2014). In particular, mononucleate macrophages or activated lymphocytes were predominant over other blood cell types (Figure 5), whereas granulocytes were more abundant than agranulated cells in blood (Figure 7). Wound exudate, which contained fibrin, was abundant in the cauterized blastemas, likely due to plasma extravasation following cauterization. The removal of the exudate likely generated intense inflammation in the blastema, leading to scarring (Alibardi, 2013).

Most lymphocytes contained sparse ribosomes and lacked endoplasmic reticulum vesicles, suggesting they were T-cells (Janossy et al., 1972). However, due to some uncertainty in the discrimination between macrophages and activated lymphocytes or between activated basophil and eosinophil granulocytes, the present quantitative data on white cell types should be considered with some caution. In the average sections of the blastema or blood, cells appeared in their active, often motile condition, and their shape and surface were distorted and not optimal for unbiased morphological identification. Furthermore, the plane of the randomly cut sections may also have intercepted cells in a cytoplasmic or nuclear region that were tangential to the main cellular area, making identification more problematic. Some studies have also indicated that lymphocytes in fish, amphibians, and some reptiles possess active phagocytosis, making the distinction with macrophages even more difficult (Li et al., 2006; Zimmerman et al., 2010). Despite these shortcomings, the present study clearly showed that significant increases in the number of granulocytes, lymphocytes, and particularly macrophages in the blastema and blood were associated with scarring.

The present study indicated that an inflammatory reaction involving an average of 15% of white blood cells invading the blastema was strong enough to limit or impair tail regeneration in lizards. Therefore, it is not surprising that the healing limb of *Podarcis muralis* at 11–12 d of regeneration after amputation, where inflammation includes over 50% of white blood cells in comparison to the total cell types present in a forming blastema (Alibardi, 2010b, 2016; 50.5%±2.4% in three quantified cases), gives rise to a scar. The main proportion of immune cells, about 70% of the total, consisted of macrophages/lymphocytes of difficult specific identification. The increase in basophils/eosinophils and macrophages/lymphocytes detected in the present study in scarring tails indicated that these cells were likely associated with the formation of scarring connective tissue. However, direct stimulation of myofibroblasts and fibrocytes in lizards from these cells was not shown. The differentiation of the latter cells can give rise to a scarring blastema during the fourth regeneration in a cauterized tail stump (Alibardi, 2010a, 2013) or in the cauterized blastema (present study).

Numerous studies on mammalian and non-mammalian vertebrates have shown that inflammation, especially chronic inflammation, is detrimental to regeneration (Ferguson & O’Kane, 2004; Leavitt et al., 2016; Mescher et al., 2013). However, the type of injury can evocate different types of inflammatory cells, in particular macrophages, some of which (healing or M2) actually favor regeneration in amphibians (Godwin & Rosenthal, 2014), fish (Petrie et al., 2014), and mammals (Hesketh et al., 2017; Simkin et al., 2017). The best regenerators among vertebrates, urodele amphibians, and also many fish, possess low inflammation and immune responses after injury, supporting the hypothesis that immune efficiency is the main obstacle to organ regeneration in amniotes. Another clear indication of the negative effect of the immune system on organ regeneration is present in anuran amphibians, which are more terrestrially-adapted than many urodeles (Mescher et al., 2013, 2017). After metamorphosis, the immune response is enhanced, and the regeneration capability is lost in frogs, toads, and some terrestrial salamanders (Mescher et al., 2013, 2017; Scadding, 1977, 1981). Evolution and potentiation of the adaptive immune system are correlated with the almost complete loss of organ regeneration in endothermic amniotes. The strong inflammation elicited in both avian and mammalian organs after injury leads to rapid resolution and variable degree of scarring (Ferguson & O’Kane, 2004; Hesketh et al., 2017). It is likely that after limb amputation or cauterization in lizards, traumatic events that induce considerable inflammation, the type of macrophages and lymphocytes activated are pro-inflammatory (M1), leading to scar formation.

The present quantitative ultrastructural study supports the hypothesis that the immune system is likely the main cause of failure of tissue and organ regeneration in lizards, and in amniotes in general (Alibardi, 2014). Future follow-up studies on this topic are required to demonstrate whether inflammatory cells respond to general stimulation after intense and persistent tissue damage or if macrophages and lymphocytes can specifically attach to mesenchymal, ependymal, and epidermal cells of the regenerating blastema.
